# How Smart Manufacturing Can Help Combat the COVID-19 Pandemic

**DOI:** 10.3390/diagnostics11050885

**Published:** 2021-05-17

**Authors:** Yun-Siang Lin, Chao-Min Cheng, Chen-Fu Chien

**Affiliations:** 1 Department of Industrial Engineering and Engineering Management, National Tsing Hua University, Hsinchu 30013, Taiwan; yunsianglin@gapp.nthu.edu.tw; 2 Institute of Biomedical Engineering, National Tsing Hua University, Hsinchu 30013, Taiwan; 3 Artificial Intelligence for Intelligent Manufacturing Systems (AIMS) Research Center, Ministry of Science and Technology, Hsinchu 30013, Taiwan

The coronavirus pandemic (COVID-19) caused by severe acute respiratory syndrome–coronavirus 2 (SARS-CoV-2) has threatened public health and caused tremendous social and economic losses [1]. Since effective treatment alternatives are lacking and infection is increasingly widespread, vaccine development, and smart manufacturing of effective vaccines to conquer this pandemic are vital. A lipid nanoparticle-encapsulated messenger RNA (mRNA-LNP) vaccine that can be swiftly developed and manufactured may efficiently meet unprecedented globalized vaccine demands [2]. An LNP vaccine manufactured using a microfluidic system offers distinct advantages of scalability, reproducibility, and rapid preparation time [3]. Although mass production of a vaccine can reduce manufacturing costs and fulfill large-scale urgent demand, vaccine safety remains a primary concern since problematic vaccines may be life-threatening. However, little research has been done to address the quality and suitability of a mass-produced mRNA-based vaccine for smart manufacturing. To ensure vaccine quality, safety, reliability, and yield, we may leverage approaches used in other high-tech manufacturing fields [4], such as wafer fabrication, that use precisely intelligent solutions for advanced quality control (AQC), advanced process control (APC), and advanced equipment control (AEC). Manufacturing excellence solutions employed in the semiconductor manufacturing field may be employed to enhance the quality of vaccine bio-manufacturing processes.

## 1. Smart Manufacturing and R2R Control for COVID-19 Vaccine Manufacturing

Semiconductor manufacturing is capital intensive, lengthy, and complicated processes are required for high precision products, while the field has seen unparalleled growth in the past few decades for continuous migration of advanced technology nodes [5]. Due to high demand and rigorous control demands, process and quality control has become increasingly difficult for nano technology nodes. Novel approaches for AQC, APC, and AEC have been developed to control production and enhance yield, including prognostic and health management (PHM), run-to-run (R2R) control, fault detection and classification (FDC) tools for analyzing large amounts of data, such as ex situ metrology data and/or in situ monitoring tool-level data, to improve process effectiveness and enhance quality [6,7,8]. R2R control approaches, including feed-forward, feedback and feed-forward/feedback controls, have been developed to proactively deal with process shifts between machine runs [9]. The feed-forward R2R controller requires metrology results from the previous process step to compensate for process variation within the previous process, while the feedback R2R controller uses the metrology value from the previous run to modify the parameter settings for the subsequent run. The feed-forward/feedback controller adapts values from the last process and the previous run to manipulate process parameters. However, metrology delay is an inevitable problem for R2R control using convolutional metrology methods. Virtual metrology (VM) technology that can adapt previous real process values to predict metrology values is viewed as the solution for metrology delay, since it can virtually predict the metrology value within a few seconds to provide the information to support real-time decision. Indeed, Industry 4.0 spurs the needs for advanced data collection and analytics technologies such as the Internet of Things (IoT), cloud computing, artificial intelligence, big data analytics, and the Cyber Physical System (CPS) that enables a more efficient and intelligent approach for AQC/APC/AEC [10]. With multiple sensors installed on machines, massive engineering and manufacturing data can be automatically recorded to derive valuable information through big data analytics and artificial intelligence technologies to empower smart manufacturing. Vaccine manufacturers can benchmark and leverage the advantages of Industry 4.0 technologies and AQC/APC/AEC solutions from industrial manufacturing to enable mass production and reduce quality variations in mRNA-based vaccines (not limited to only mRNA-based vaccines, there is potential for manufacturing other types of vaccines as well).

## 2. Concept of R2R Control for mRNA-LNP Vaccine Manufacturing

**Figure 1:**
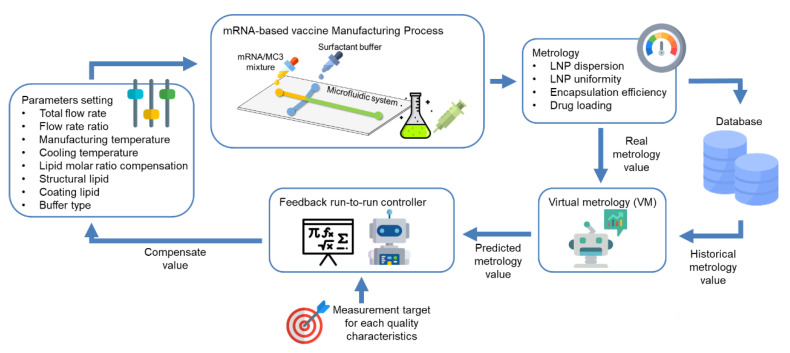
VM and R2R framework for mRNA-LNP vaccine manufacturing through using a microfluidic system (not limited to mRNA vaccines, potential for manufacturing other types of vaccines as well).

To ensure vaccine safety and effectiveness, a number of quality characteristics should be considered during manufacturing: (1) LNP dispersion, which relates to vaccine stability; (2) LNP uniformity, which affects structure and size; and (3) LNP encapsulation efficiency and drug loading. The controllable process parameters for vaccine manufacturing can be categorized into two types, namely operating parameters and formula parameters. The operating parameters determine the settings for microfluidic system, including total flow rate, flow rate ratio, manufacturing temperature, and cooling temperature. The formula parameters that come from the mRNA mixture and surfactant buffer involve lipid molar ratio compensation, structural lipid, coating lipid, and buffer type. Since microfluidic mRNA-based vaccine manufacturing contains only a single process step without measurement results from the previous step, the feedback R2R schema that returns metrology information may be employed as a solution. Thus, VM can be adapted to enhance the R2R controller reaction speed for a vaccine manufacturing microfluidic system as illustrated in Figure 1. First, the operational and formula recipe parameters are measured and recorded in a database for the corresponding quality characteristics after producing the LNP-delivered mRNA. Then, VM uses the real quality measurements and historical metrology values to fine tune the prediction model to provide a more accurate predicted metrology value. The feedback R2R controller retrieves the predicted metrology value and the measurement target for each quality characteristics to estimate the compensating value of the parameters setting to reduce process shift and variation for each run. With the proposed engineering-based R2R controller, vaccine quality can become more stabilized for mass production. Through mass production and the proposed R2R control framework, the quantity and the effectiveness of manufactured COVID-19 vaccine could improve, easing vaccine shortage and distribution issues, and helping the global society recover from the COVID-19 pandemic.
